# Supplementary material for: Splicing factor SRSF1 deficiency in the liver triggers NASH-like pathology and cell death
Source: Nat Commun. 2023 Feb 9;14:551. doi: 10.1038/s41467-023-35932-3 (PMC9911759; doi:10.1038/s41467-023-35932-3)
Supplement: Supplementary file 1 — Supplementary Information [file 41467_2023_35932_MOESM1_ESM.pdf]

Supplementary Materials for

Splicing Factor SRSF1 Deficiency in the Liver Triggers NASH-like Pathology and Cell Death

Waqar Arif, Bhoomika Mathur, Michael F. Saikali, Ullas V. Chembazhi, Katelyn Toohill, You Jin Song, Qinyu Hao, Saman Karimi, Steven M. Blue, Brian A. Yee, Eric L. Van Nostrand, Sushant Bangru, Grace Guzman, Gene W. Yeo, Kannanganattu V. Prasanth, Sayeepriyadarshini Anakk, Carolyn L. Cummins, Auinash Kalsotra

*Corresponding author. Email: kalsotra@illinois.edu (A.K.)

Supplementary Figures

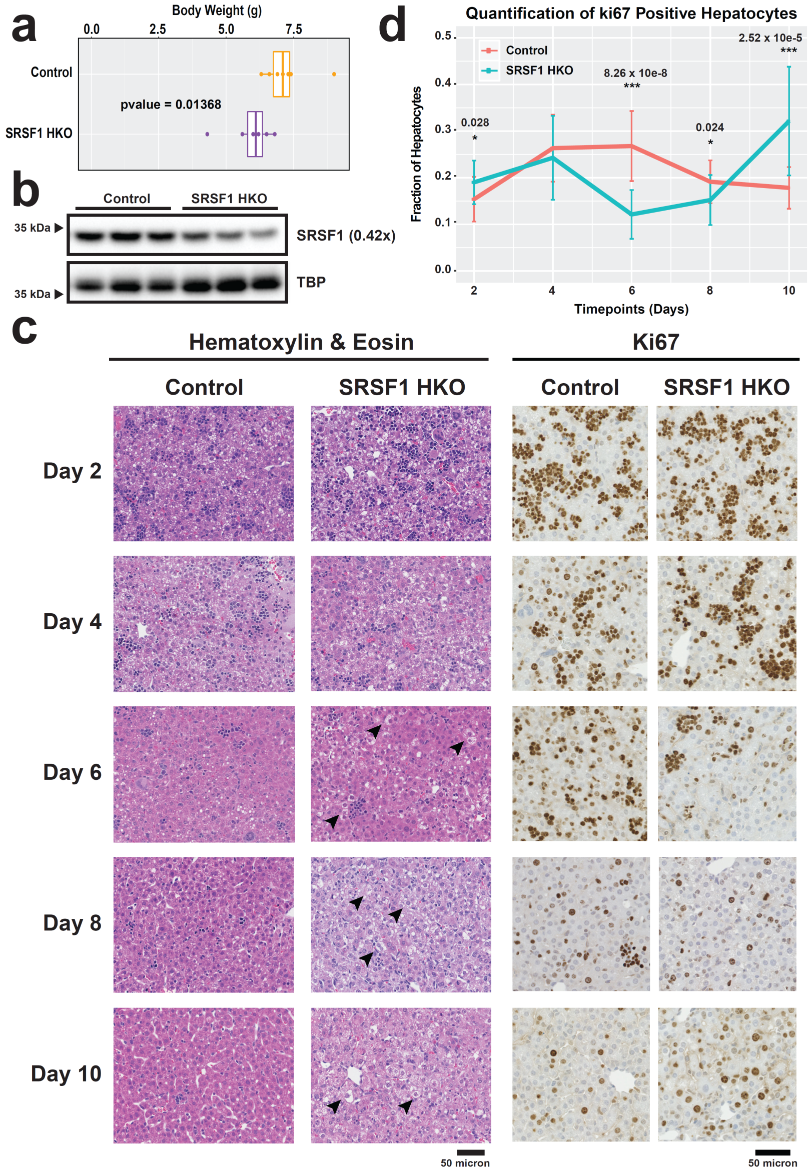

Supplementary Figure 1. Phenotypic characterization of SRSF1 HKO at post-natal timepoints. (a) Total body weight measurements at post-natal day 10 (PN10) of SRSF1 HKO and Control mice (n = 6 per group). A two-tailed Welch two-sample t-test was performed. **(b)** Representative western blot image for relative abundance measurements of SRSF1 in hepatocytes isolated from PN10 SRSF1 HKO and Control mice with TBP as the loading control. Value in parentheses signifies abundance fold change in SRSF1 HKO relative to Control. **(c)** Hematoxylin & Eosin *(Left)* and Ki67 antigen, a proliferation marker, immunohistochemical *(Right)* staining of liver sections from SRSF1 HKO and Control mice at the indicated timepoint. Black arrows indicate ballooning necrotic hepatocytes. **(d)** Quantification of the fraction of Ki67-positive hepatocytes from staining images for each group and timepoint (n = 3 biologically independent animals per group, 6 fields per replicate). Values are displayed as mean ± SD. A two-way ANOVA analysis was performed with Tukey multiple comparisons of means. Significant differences are denoted by "*" with the p-value listed above.

Age	Group	Replicate	Steatosis (0-3)
Post-Natal Day 2	Control	1	3
	Control	2	1
	Control	3	2
	**Average**		**2.00**
	SRSF1 KO	1	3
	SRSF1 KO	2	1
	SRSF1 KO	3	3
	**Average**		**2.33**
Post-Natal Day 6	Control	1	2
	Control	2	1
	Control	3	1
	**Average**		**1.33**
	SRSF1 KO	1	2
	SRSF1 KO	2	2
	SRSF1 KO	3	1
	**Average**		**1.67**
Post-Natal Day 10	Control	1	0
	Control	2	0
	Control	3	0
	**Average**		**0.00**
	SRSF1 KO	1	2
	SRSF1 KO	2	1
	SRSF1 KO	3	1
	SRSF1 KO	4	2
	SRSF1 KO	5	1
	**Average**		**1.4**

Supplementary Table 1. Histological steatosis scoring of post-natal livers. Scores range from values of 0 to 3, which correspond to <5% (0), 5% - 33% (1), >33% - 66% (2), or >66% (3).

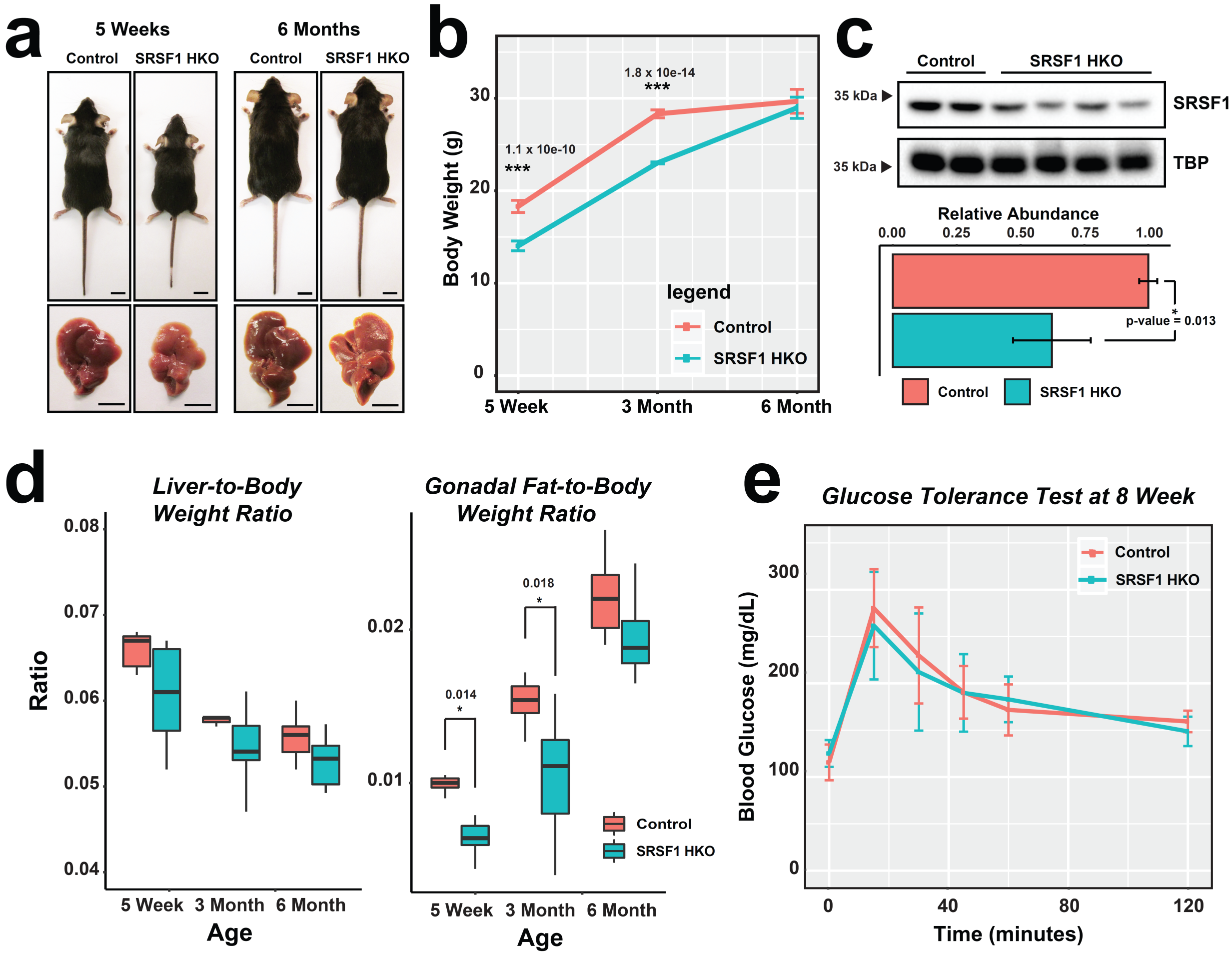

Supplementary Figure 2. Phenotypic differences in SRSF1 HKO mice progressively normalize to Control mice. (a) Gross appearance of control and SRSF1 HKO mice and livers at 5 weeks and 6 months timepoints. Scale bar = 1 cm. **(b)** Body weight growth curve of controls and SRSF1 HKO mice. **(c)** Relative SRSF1 protein abundance quantification in SRSF1 HKO mice at 5 weeks using western blot analysis. **(d)** Box plot of liver-to-body weight and gonadal fat-to-body weight ratios in control and SRSF1 HKO mice at indicated timepoints during adult development. A two-way ANOVA analysis was performed with Tukey multiple comparisons of means. Significant differences are denoted by "*" with the p-value is listed above. **(e)** Intraperitoneal glucose tolerance test of control and SRSF1 HKO mice at 8 weeks of age (n = 10 and 11 for control and SRSF1 HKO, respectively). The area under curve p-value = 0.759. Values are displayed as mean ± SD. Two-tailed unpaired *t*-test was used to determine the significance between the 2 groups.

Mouse Model	Age	Group	Gender	Steatosis (0-3)	Lobular Inflammation (0-3)	Hepatocellular Ballooning (0-2)	Total NAS Score	Fibrosis Score (0-4)
Transgenic SRSF1 HKO	1 Month	Control	Male	1	0	1	2	0
		Control	Male	0	0	1	1	1
		Control	Male	0	1	0	1	1
		Control	Male	0	0	1	1	2
		Control	Female	0	1	0	1	1
		Control	Female	0	1	0	1	2
		Control	Female	1	1	2	4	2
		Control	Female	0	1	1	2	2
		Average		0.25	0.625	0.75	1.625	1.375
		SRSF1 HKO	Male	2	2	2	6	4
		SRSF1 HKO	Male	2	1	2	5	3
		SRSF1 HKO	Male	3	1	2	6	4
		SRSF1 HKO	Male	2	2	2	6	4
		SRSF1 HKO	Female	1	2	2	5	4
		SRSF1 HKO	Female	1	2	2	5	4
		SRSF1 HKO	Female	1	2	2	5	4
		SRSF1 HKO	Female	2	2	2	6	3
		Average		1.75	1.75	2	5.5	3.75
		**Δ Score**		**+1.5**	**+1.125**	**+1.25**	**+3.875**	**+2.375**
		**Mann-Whitney**	**U-Value (13)**	**3**	**5**	**4**	**0**	**0**
		**U Test**	**p-value**	**0.00278**	**0.00544**	**0.00386**	**0.00094**	**0.00094**
	3 Month	Control	Male	0	1	0	1	1
		Control	Male	0	1	0	1	1
		Control	Male	0	1	2	3	2
		Control	Male	0	1	2	3	1
		Control	Female	0	1	0	1	1
		Control	Female	0	0	2	2	1
		Average		0.00	0.83	1.00	1.83	1.17
		SRSF1 HKO	Male	0	1	1	2	3
		SRSF1 HKO	Male	1	1	2	4	4
		SRSF1 HKO	Male	0	1	2	3	4
		SRSF1 HKO	Male	0	2	2	4	4
		SRSF1 HKO	Male	1	1	2	4	3
		SRSF1 HKO	Female	0	2	2	4	4
		SRSF1 HKO	Female	0	1	2	3	3
		Average		0.29	1.29	1.86	3.43	3.57
		**Δ Score**		**+0.28**	**+0.45**	**+0.86**	**+1.60**	**+2.40**
		**Mann-Whitney**	**U-Value (6)**	**15**	**12.5**	**12**	**4.5**	**0**
		**U Test**	**p-value**	**> 0.05**	**> 0.05**	**> 0.05**	**0.0220**	**0.00338**
	6 Month	Control	Male	0	0	1	1	1
		Control	Male	0	1	1	2	2
		Control	Male	0	1	1	2	2
		Control	Male	0	1	1	2	1
		Control	Female	0	1	1	2	2
		Control	Female	0	1	1	2	2
		Average		0.00	0.83	1.00	1.83	1.67
		SRSF1 HKO	Male	2	1	2	5	4
		SRSF1 HKO	Male	0	1	2	3	3
		SRSF1 HKO	Male	1	2	2	5	4
		SRSF1 HKO	Male	1	0	2	3	3
		SRSF1 HKO	Female	1	1	1	3	3
		SRSF1 HKO	Female	0	1	1	2	4
		SRSF1 HKO	Female	2	1	2	5	4
		SRSF1 HKO	Female	0	1	1	2	4
		Average		0.88	1.00	1.63	3.50	3.63
		**Δ Score**		**+0.88**	**+0.17**	**+0.63**	**+1.67**	**+1.96**
		**Mann-Whitney**	**U-Value (8)**	**9**	**20.5**	**9**	**5**	**0**
		**U Test**	**p-value**	**> 0.05**	**> 0.05**	**> 0.05**	**0.0168**	**0.00244**
Acute SRSF1 HKO, viral induction model	2 Week	Control (GFP)	Female	0	0	0	0	0
		Control	Male	0	0	1	1	0
		Control	Male	0	0	1	1	0
		Control	Male	0	1	1	2	1
		Average		0	0.25	0.75	1	0.25
		acSRSF1 HKO	Male	0	1	2	3	1
		acSRSF1 HKO	Male	1	1	2	4	1
		acSRSF1 HKO	Male	1	2	0	3	1
		acSRSF1 HKO	Female	0	0	1	1	1
		Average		0.5	1	1.25	2.75	1
		**Δ Score**		**+0.5**	**+0.75**	**+0.50**	**+1.75**	**+0.75**
	4 Week	Control	Female	0	1	1	2	1
		Control	Male	0	1	1	2	1
		Control	Female	0	1	1	2	1
		Control	Female	0	0	0	0	1
		Control	Male	0	1	0	1	1
		Average		0	0.8	0.6	1.4	1
		acSRSF1 HKO	Male	3	2	1	6	2
		acSRSF1 HKO	Male	2	2	2	6	2
		acSRSF1 HKO	Male	1	2	1	4	1
		acSRSF1 HKO	Female	0	2	1	3	0
		acSRSF1 HKO	Male	3	2	1	6	2
		acSRSF1 HKO	Male	3	2	1	6	2
		Average		2.00	2.00	1.17	5.17	1.50
		**Δ Score**		**+2.00**	**+1.20**	**+0.57**	**+3.77**	**+0.50**
		**Mann-Whitney**	**U-Value (3)**	**2.5**	**0**	**7.5**	**0**	**7.5**
		**U Test**	**p-value**	**0.0285**	**0.0084**	**> 0.05**	**0.00804**	**> 0.05**

Supplementary Table 2. Blinded NASH Scoring by pathologists of histological liver sections of SRSF1 HKO and acSRSF1 HKO mice. The Kleiner histological NAFLD activity scoring (NAS) system was used. A two-tailed Mann-Whitney U Test was performed to determine statistical significance. *Statistical testing was not performed at the 2-week timepoint in the acute SRSF1 HKOs due to insufficient sample size.

Serum Parameter	Age	Control	SRSF1 HKO	P-Value	Direction
**Blood Glucose (mg/dL)**	1 Month	118.7 ± 3.8	121.7 ± 6.8	0.2925	N.S.
	3 Month	129.4 ± 4.6	118.7 ± 9.3	0.0242	Down
	6 Month	138.7 ± 19.7	125.0 ± 20.6	0.2186	N.S.
**ALT (U/L)**	1 Month	19.2 ± 8.5	231.1 ± 58.7	0.0001	Up
	3 Month	13.9 ± 5.6	27.8 ± 12.1	0.0474	Up
	6 Month	9.1 ± 5.2	15.4 ± 5.6	0.1005	N.S.
**AST (U/L)**	1 Month	43.1 ± 31.2	225.2 ± 39.4	0.0001	Up
	3 Month	25.7 ± 7.8	89.8 ± 17.0	0.0002	Up
	6 Month	41.1 ± 10.5	42.5 ± 18.5	0.9018	N.S.
**Triglyceride (mg/dL)**	1 Month	80.4 ± 13.7	65.5 ± 2.9	0.0277	Down
	3 Month	143.7 ± 10.6	100.5 ± 12.6	0.0000	Down
	6 Month	115.9 ± 10.7	128.9 ± 22.1	0.2020	N.S.
**Cholesterol (mg/dL)**	1 Month	119.2 ± 22.7	90.2 ± 9.9	0.0343	Down
	3 Month	96.7 ± 22.4	120.6 ± 17.3	0.0350	Down
	6 Month	103.1 ± 8.0	121.5 ± 21.0	0.0612	N.S.

Supplementary Table 3. Measurement of serum metabolic and liver function markers in SRSF1 HKO and Control mice at the indicated ages. Mice were fasted for 10 hours prior to collection of serum (n = 7 per group). P-values were calculated using a two-tailed Welch two sample t-test with Bonferroni correction.

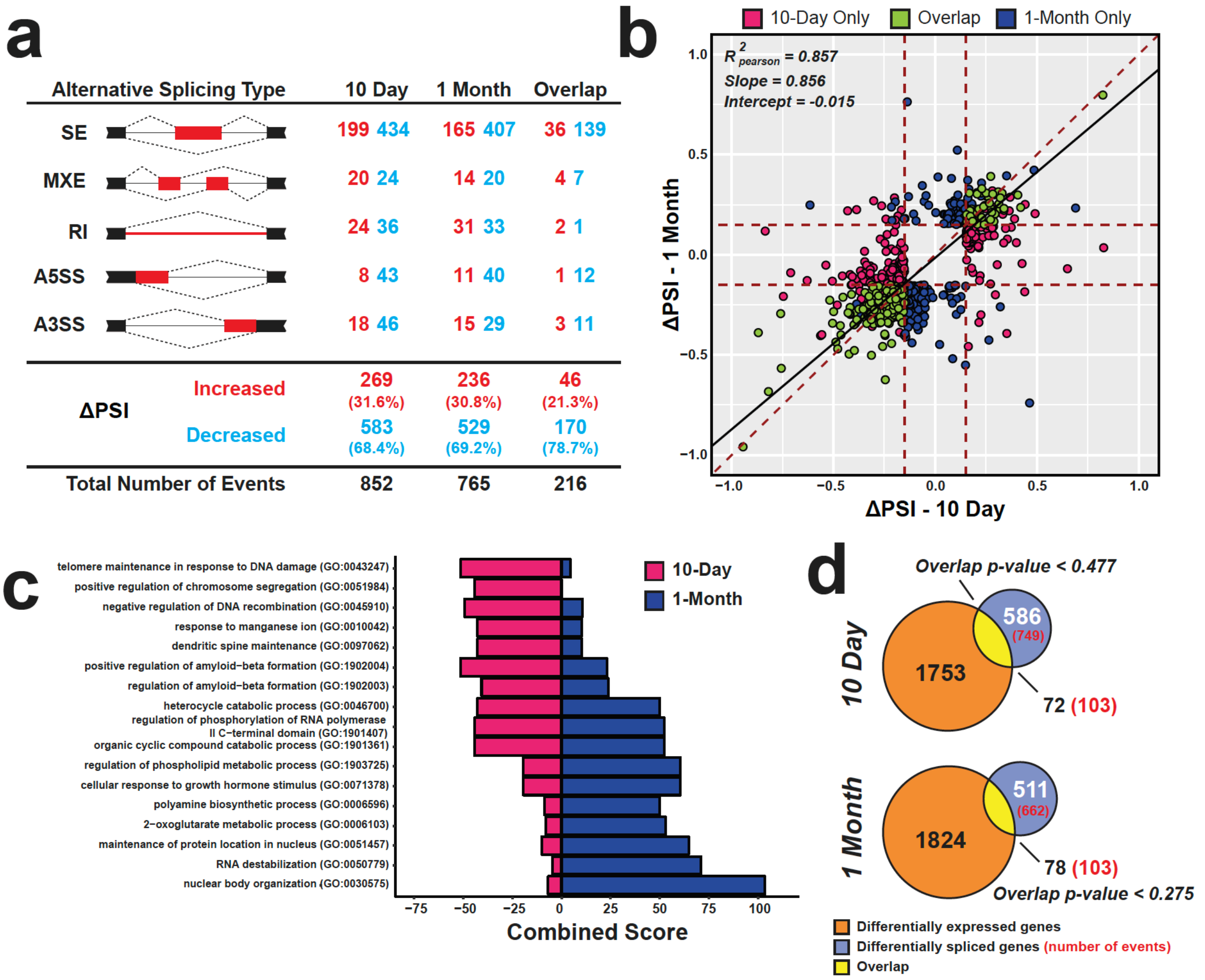

Supplementary Figure 3. RNA-seq analysis of SRSF1 HKO reveals extensive changes in exon usage. (a) Breakdown of the number of differentially spliced exons (FDR < 0.10, junction read counts ≥ 10, and difference in Percent Spliced Index |ΔPSI| > 15%) by event type in 10-day and 1-month SRSF1 HKO mice. Numbers in red signify increased inclusion while blue signify decreased inclusion in SRSF1 HKO. SE, skipped exon; MXE, mutually exclusive exons; RI, retained intron; A5SS, alternative 5' splice site; A3SS, alternative 3' splice site. **(b)** Scatter plot showing the distribution of ΔPSI values for differentially spliced exons in 10-day and 1-month SRSF1 HKO mice. **(c)** Enrichr gene ontology analysis using genes with differentially spliced exons in 10-day and 1-month SRSF1 HKO. Larger combined score values signify greater enrichment. **(d)** Venn diagrams depicting the overlap of genes with differential expression and splicing at each timepoint.

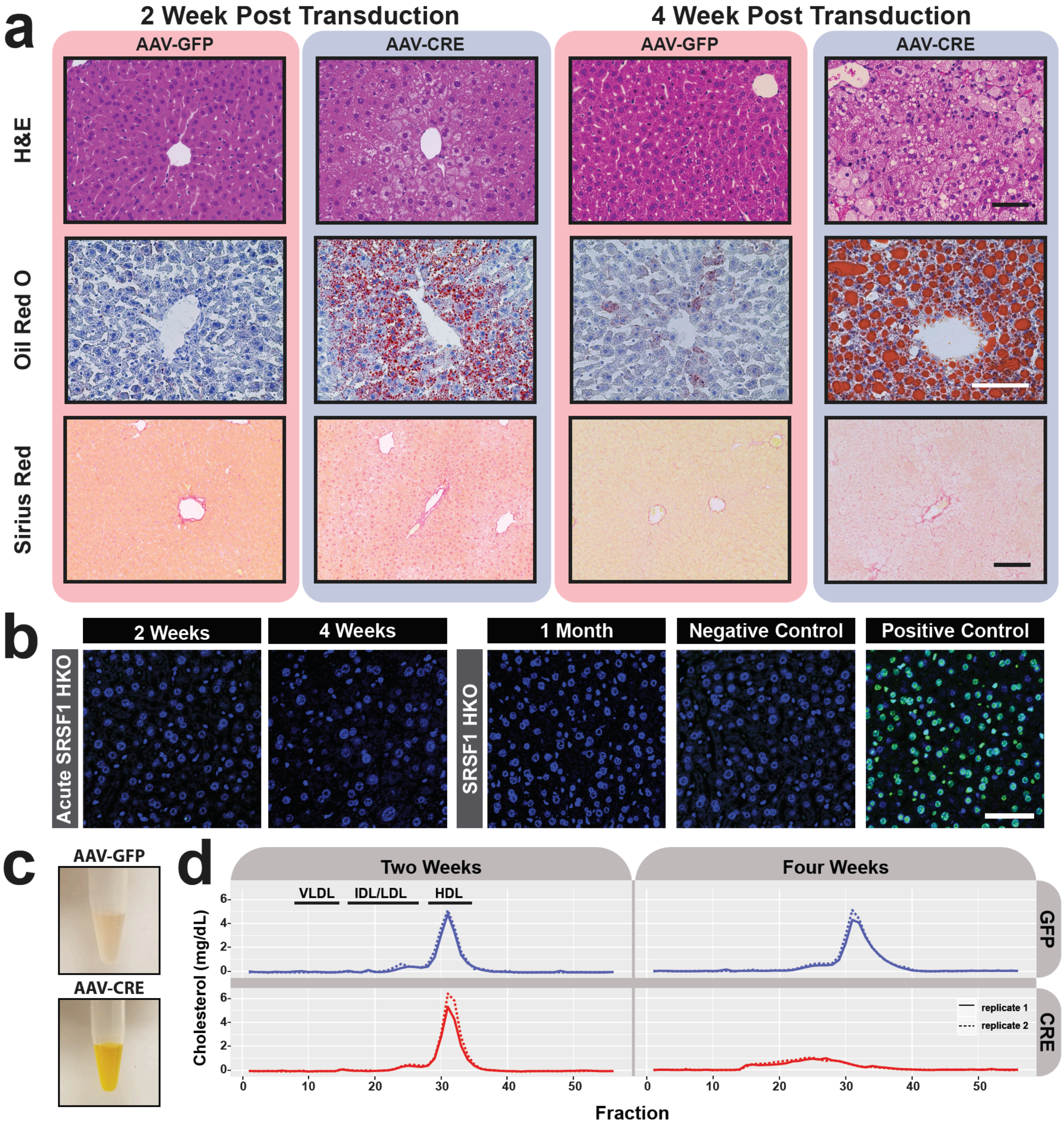

Supplementary Figure 4. Acute loss of SRSF1 in adult hepatocytes recapitulates the SRSF1 HKO phenotype. (a) Representative images of H&E, Oil Red O (red, neutral lipids), and Sirius Red (red, collagen) histological staining of liver tissue harvested from control and acSRSF1 HKO mice at the indicated timepoints (n = 6 per group). Scale bar = 100 microns. **(b)** Representative images of TUNEL staining for apoptosis detection in acSRSF1 HKO mice at 2 and 4 weeks and SRSF1 HKO mice at 1 month. Green nuclei indicate apoptotic cells. Scale bar = 100 microns. **(c)** Representative images of serum collected from control (AAV-GFP) and acSRSF1 HKO (AAV-CRE) 4 weeks post-viral transduction of SRSF1$^{flox/flox}$ mice. **(d)** Serum lipoprotein particle fractionation from 6-hour fasted control and acSRSF1 HKO mice. Each replicate consists of serum pooled from 2 biological replicates.

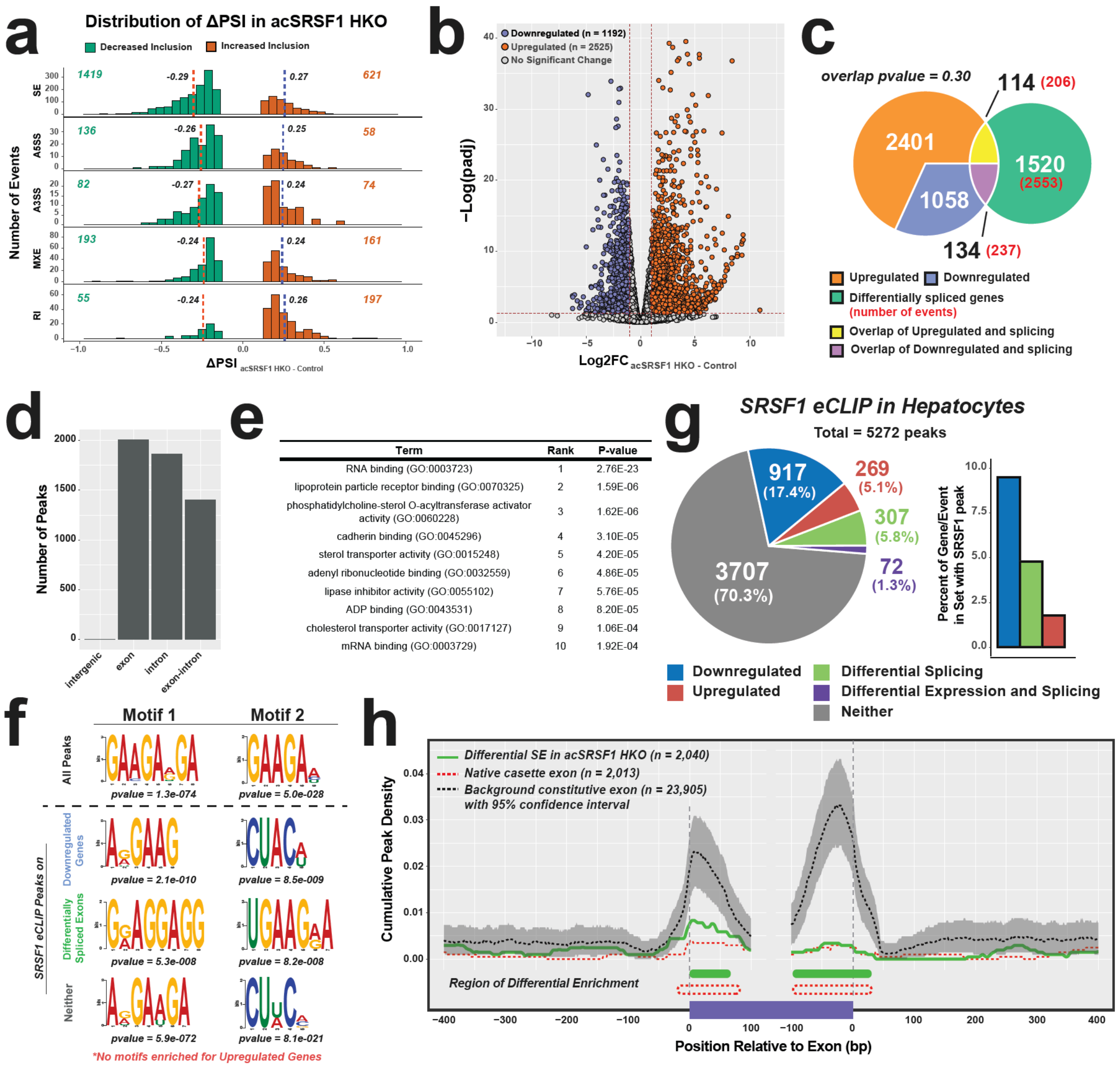

Supplementary Figure 5. Hepatocytes from acSRSF1 HKO display widespread transcriptome defects. **(a)** Histograms showing the distribution of ΔPSI values of differentially spliced exons in acSRSF1 HKO with respect to controls from RNA-seq (n = 3 replicates per group). Distributions are separated by the type of alternative exon event. Dotted vertical lines indicated the median of distribution. **(b)** Volcano plot showing changes in mRNA abundance in acSRSF1 HKO. **(c)** Venn diagrams showing overlap of genes with differential expression and splicing in acSRSF1 HKO. Overlap significance was determined using a hypergeometric test. **(d)** Breakdown of the number of SRSF1 eCLIP binding peaks located at the indicated pre-mRNA region. **(e)** Gene ontology analysis of the genes with SRSF1 binding peaks using Enrichr. The Enrichr application performs a Fisher exact test with correction for multiple testing. **(f)** Top two enriched motifs for all SRSF1 eCLIP binding peak regions and for peaks associated with regulated gene sets. Motif enrichment analysis and significant testing was performed using the STREME analysis tool. **(g)** Pie chart showing the percentage of SRSF1 binding peaks found to overlap with differentially expressed or spliced genes in acSRSF1 HKO. The bar plot on right shows the inverse relationship. **(h)** Cumulative peak density plot of SRSF1 binding

peaks on differentially spliced cassette exons (green line) found in acSRSF1 HKO, native cassette exons (red), and constitutive exons (black) as background. The background set was sampled 1,000 times to determine the mean (black dotted line) and the 95% confidence interval (gray region) of peak density at each nucleotide position. Native cassette exon set consists of exons found to be alternative in both control and acSRSF1 HKO but did not change significantly ($0.05 < PSI < 0.95$ and $|\Delta PSI| \leq 0.05$) upon SRSF1 knockout.

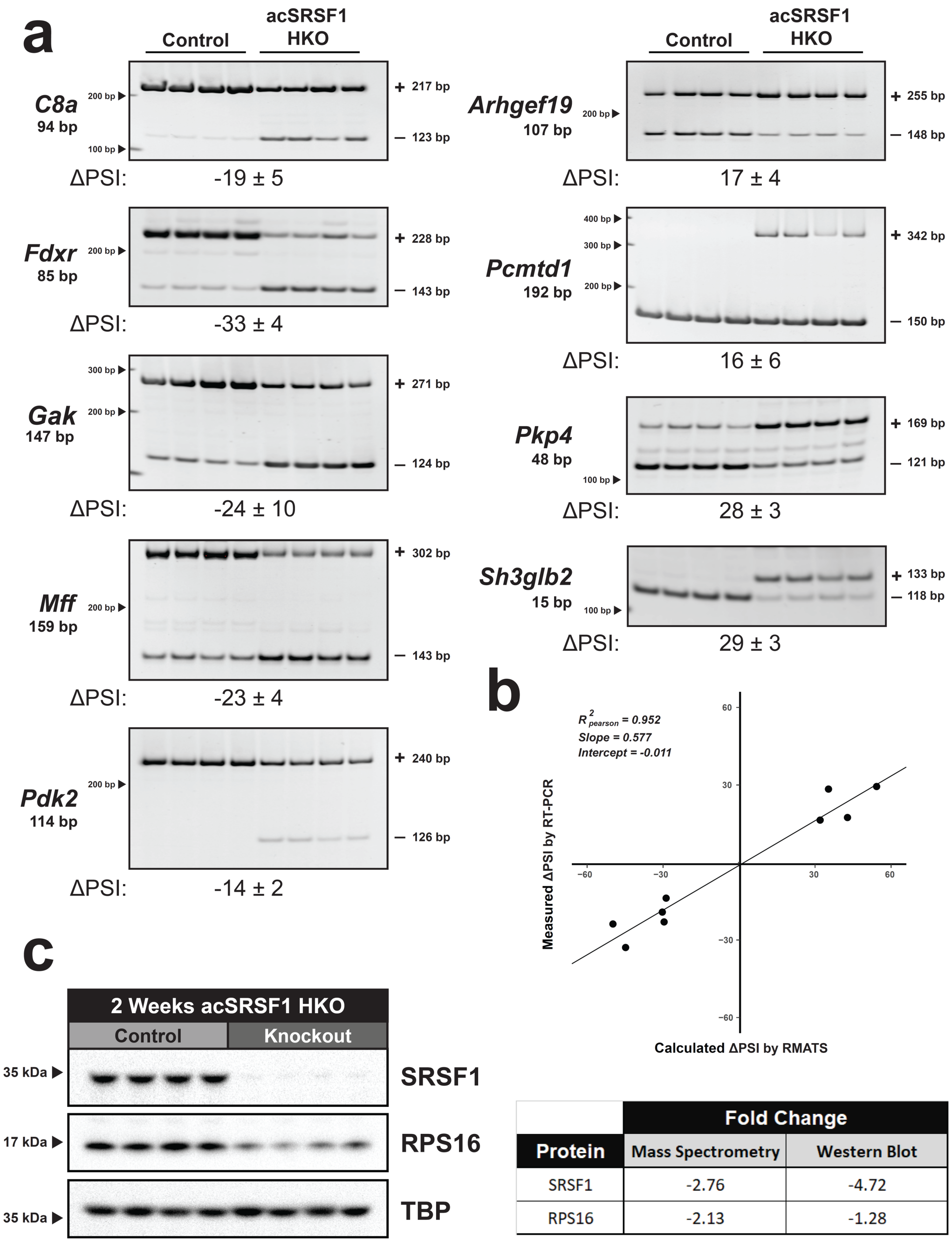

	Fold Change	
**Protein**	**Mass Spectrometry**	**Western Blot**
SRSF1	-2.76	-4.72
RPS16	-2.13	-1.28

Supplementary Figure 6. Validation of alternative splicing and proteome data. (a) RT-PCR validation of alternative splicing in transcripts sampled from the rMATS output to represent events that are increasing and decreasing in inclusion between control and acSRSF1 HKO hepatocytes. The bands corresponding to (+) indicate exon inclusion, and (-) indicate exon exclusion. n=4 **(b)** Scatter plot showing the comparison of RT-PCR and RNA-seq based ΔPSI values. **(c)** Western blots showing SRSF1 and RPS16 protein levels in hepatocytes from controls and acSRSF1 HKO mice with TBP serving as loading control n=4. Comparison of fold changes in protein abundance of SRSF1 and RPS16 based on calculated IBAQ values from mass spectrometry and western blot analysis.

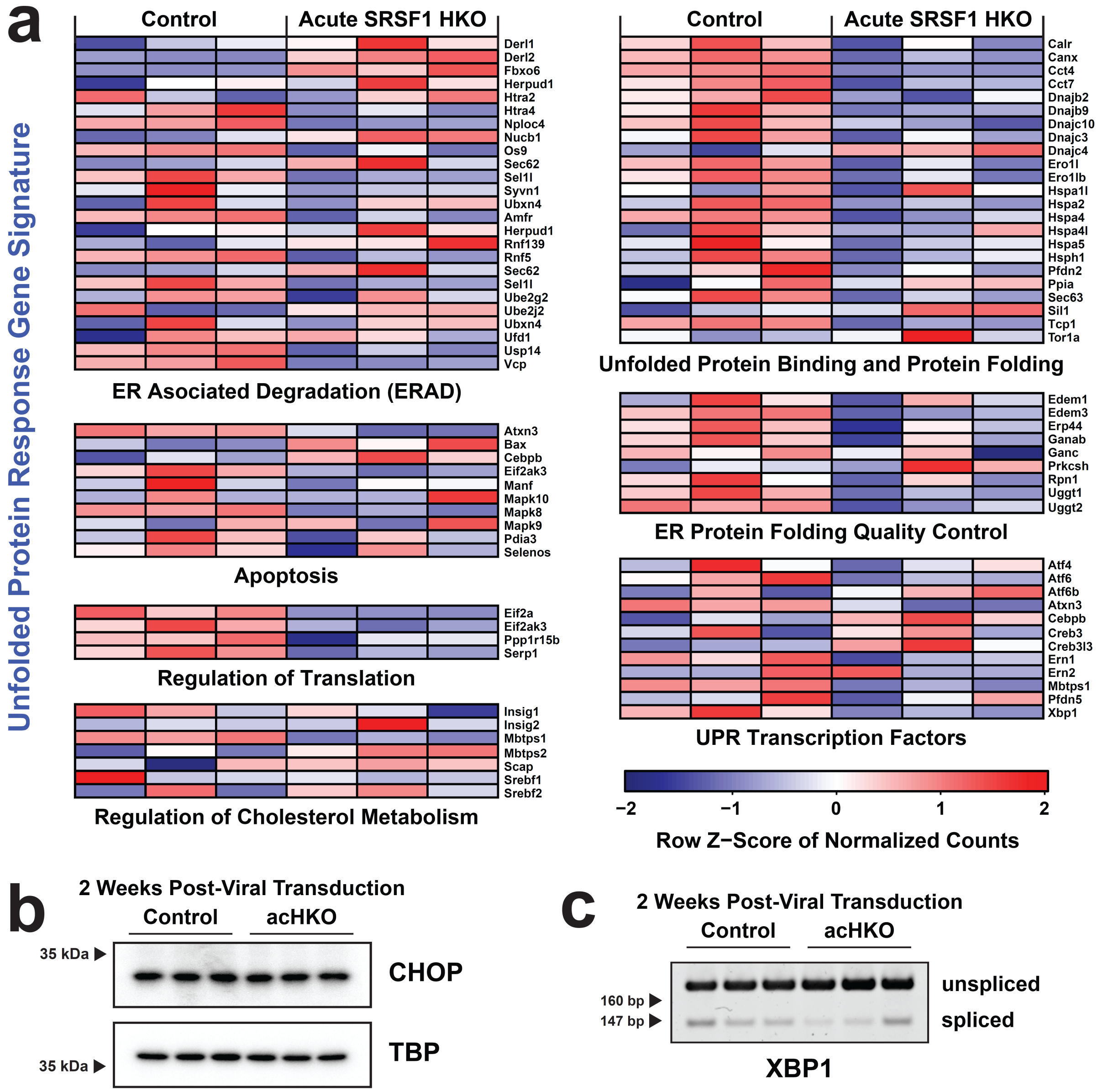

Supplementary Figure 7. Knockout of SRSF1 does not trigger the Unfolded Protein Response. (a) Heatmap showing row normalized counts for genes involved in the Unfolded Protein Response, or UPR. Each column represents a single replicate. **(b)** Western blot showing CHOP levels in controls and acSRSF1 HKO with TBP serving as the loading control. No significant difference in measured relative abundance. **(c)** XBP1 RT-PCR products showing relative levels of unspliced and spliced transcripts. No significant shift in splicing was detected between the control and acSRSF1 HKO.

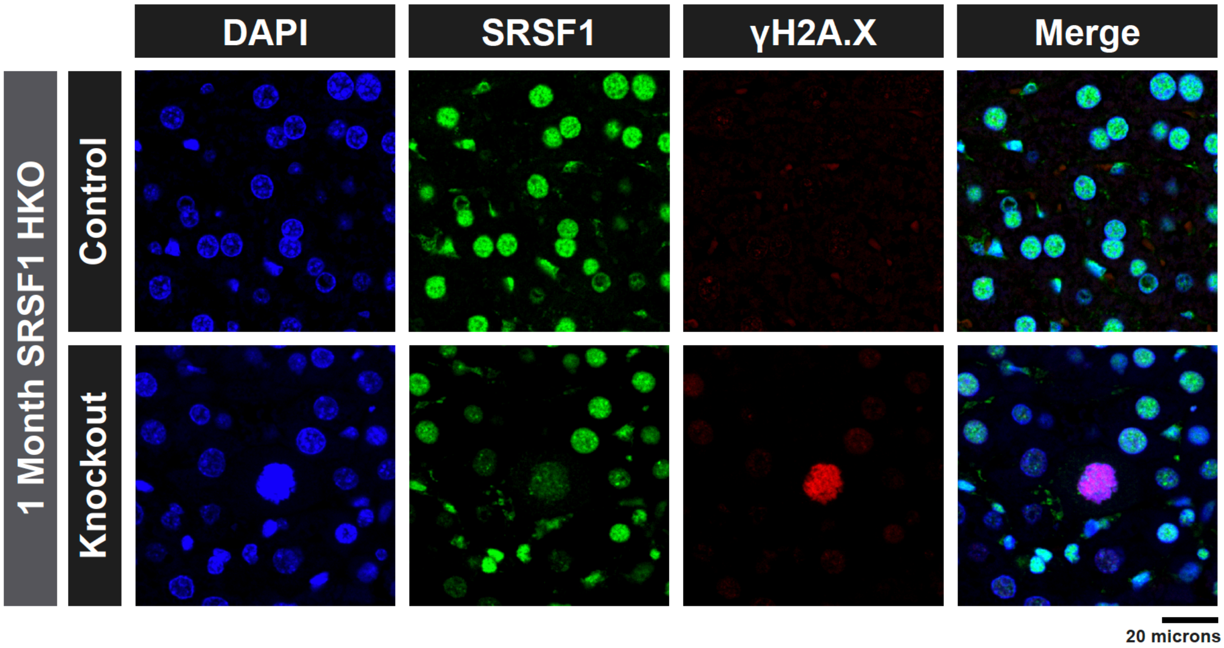

Supplementary Figure 8. Loss of SRSF1 results in DNA damage. (a) Representative immunofluorescence (IF) images of liver sections from 1-month SRSF1 HKO mice (n = 3) probed for SRSF1 (green), γH2A.x (red), a DNA damage marker, with nuclear counterstaining using DAPI (blue).

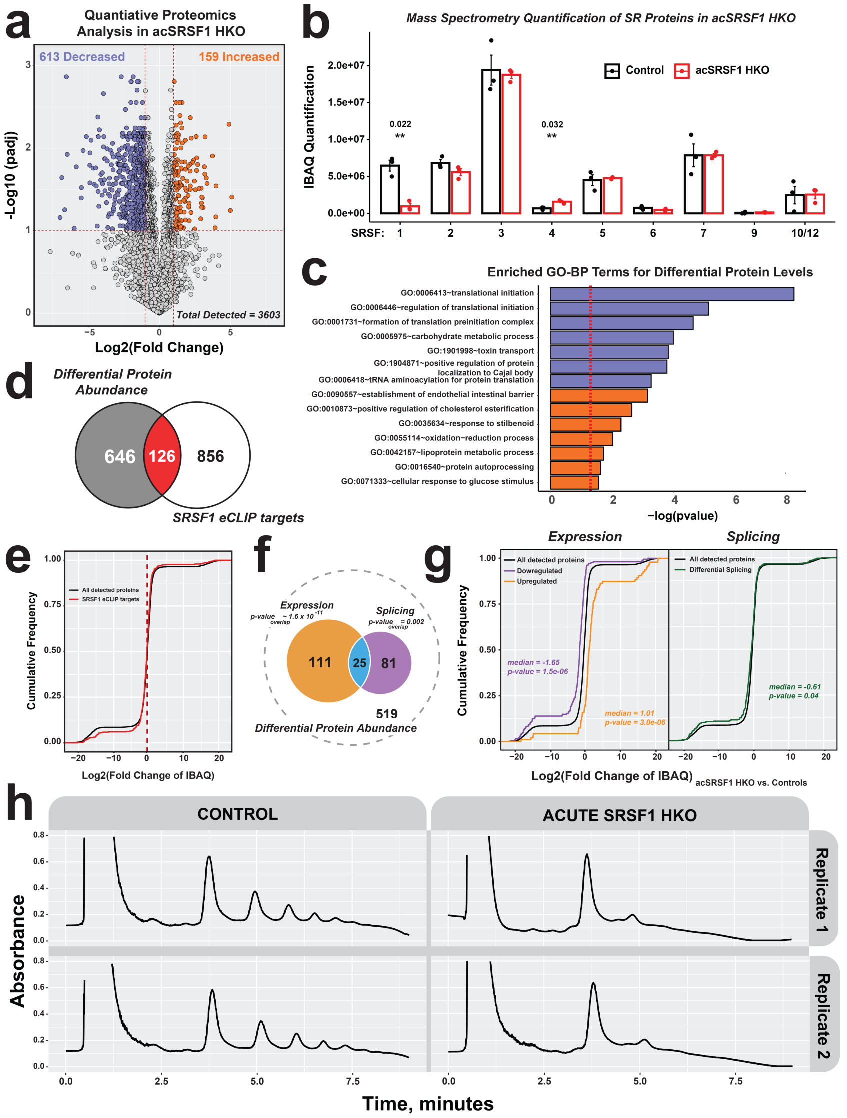

Supplementary Figure 9. Disruption of the hepatic proteome in acSRSF1 HKO. (a) Plot depicting significant changes in relative protein abundances (Log_2|IBAQ ratio| >1, adjusted p-value < 0.1) estimated by quantitative mass-spectrometry-based proteomics of control and acSRSF1 HKO (n = 3 per group). Differential protein abundance was performed using student's t-test with Bonferroni correction on iBAQ values between control and acSRSF1 HKO. **(b)** The plot of IBAQ quantification of detected SR proteins (n = 3 per group). Values are displayed as mean IBAQ values ± SEM and calculated p-values for significantly differentially expressed SR proteins. **(c)** GO analysis with computed p-values using Enrichr of differentially abundant proteins (Log_2(Fold Change) > 1, FDR < 0.10) in acSRSF1 HKO. **(d)** Overlap between differentially abundant proteins in acSRSF1 HKO and genes with SRSF1 eCLIP binding peaks. **(e)** Cumulative plot of protein fold-changes of all detected proteins (black) and subset with SRSF1 binding on associated genes. **(f)** Overlap of differentially abundant proteins with genes changing in expression or splicing with calculated overlap p-values by hypergeometric testing. **(g)** Cumulative plot of protein fold-change values for associated genes that are differentially expressed (purple and orange for down- and up-regulated, respectively) or spliced (green) in acSRSF1 HKO. A Wilcoxon rank sum test with continuity correction was performed to determine significance. **(h)** Polysome profiles in hepatocytes from control and acSRSF1 HKO at 2 weeks post-viral induction (n = 2 per group).

Knockdown in HepG2	Differential Expression (DESeq2)			Differential Splicing (rMATS)		
	Upregulated	Downregulated	Total	Included	Skipped	Total
**SRSF1**	1591	2312	3903	1999	1367	3366
**SRSF3**	834	376	1210	1087	624	1711
**SRSF5**	867	547	1414	729	667	1396
**SRSF7**	844	546	1390	433	587	1020
**SRSF9**	1272	1111	2383	476	865	1341

Supplementary Table 4. Number of genes with differential expression or exons with differential splicing after shRNA knockdown of SR genes in HepG2 from the ENCODE Project. DESeq2 and rMATS analysis was performed on ENCODE Project RNA-seq datasets for the indicated SR protein knockdown by shRNA in HepG2 cells. Differential expression and splicing were determined using parameter cut-offs as described previously.

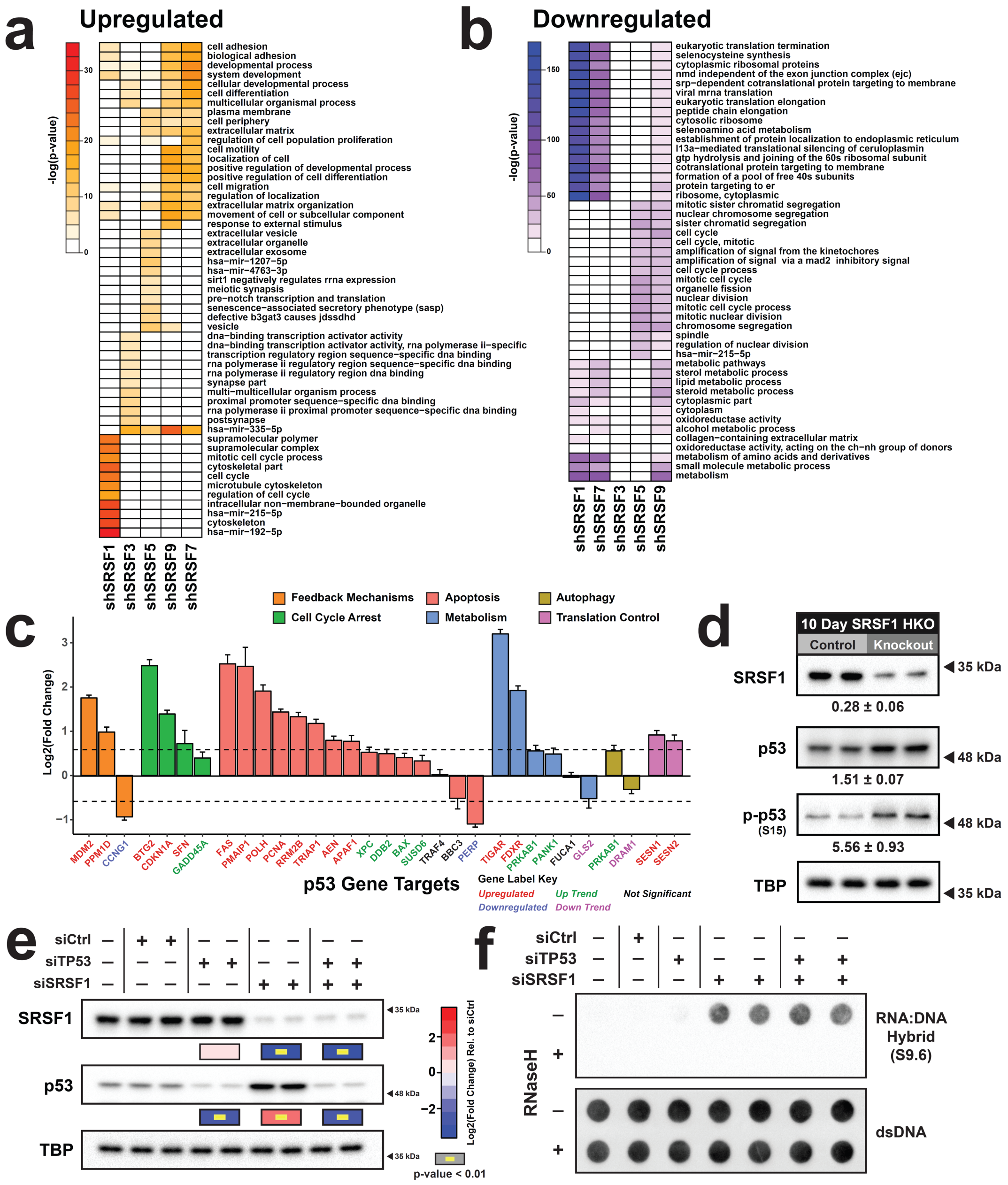

Supplementary Figure 10. Knockdown of SRSF1 in HepG2 triggers a p53 Response. Heatmaps of gene ontology enrichment terms based on -log(p-value) obtained from Enrichr for **(a)** upregulated and **(b)** downregulated genes in shRNA knockdown of SR proteins in HepG2 cells from the ENCODE Project. **(c)** Bar plot showing fold-changes determined by RNA-seq analysis for the expression of various p53 target genes in HepG2 cells after SRSF1 knockdown (n = 2 per group). The error bars represent the standard error estimated by DESeq2. The color of x-axis gene labels depicts the direction of the fold change. **(d)** Western blot of 10-day control and SRSF1 HKO mice hepatocytes (n = 4 per group). Values under blot display mean ± SD of the abundance fold change in SRSF1 HKO relative to controls. **(e)** Western blots with quantifications represented below each blot in HepG2 cells with the indicated siRNA treatment (n=4 per group). **(f)** Representative R-loop dot blot on purified DNA from HepG2 cells with the specified siRNA knockdown (n = 4 per group). RNAse H treated samples serve as a negative control, and a dsDNA dot blot confirms equal loading.

Antibody	Host Species	Type	Applications	Supplier Information, Catalog Number
anti-Mouse	Goat	Secondary, HRP	WB - 1:5000	Bio-Rad, 1721011
anti-Mouse, Dylight 488	Goat	Secondary, Flourescence	IF - 1:500	Thermo Fisher Scientific, 35503
anti-Mouse, Dylight 594	Goat	Secondary, Flourescence	IF - 1:500	Thermo Fisher Scientific, 35511
anti-Rabbit	Goat	Secondary, HRP	WB - 1:5000	Thermo Fisher Scientific, 31460
anti-Rabbit, Dylight 488	Goat	Secondary, Flourescence	IF - 1:500	Thermo Fisher Scientific, 35553
anti-Rabbit, Dylight 594	Goat	Secondary, Flourescence	IF - 1:500	Thermo Fisher Scientific, 35561
BAX	Rabbit	Primary	WB - 1:5000	Abcam, ab32503
Beta-actin	Rabbit	Primary	WB - 1:5000	Cell Signaling Technology, 8457
DNA-RNA Hybrid, Clone S9.6	Mouse	Primary	DB - 1:5000	Abcam, ab256361
ds DNA	Mouse	Primary	DB - 1:5000	Abcam, ab27156
eIF2α	Rabbit	Primary	WB - 1:5000	Cell Signaling Technology, 9722
Hnf4a	Mouse	Primary	IF - 1:500	Abcam, ab41898
Hnf4a	Rabbit	Primary	IF - 1:500	Cell Signaling Technology, 3113
Ki67	Mouse	Primary	IHC - 1:250	BD Biosciences, 550609
p53	Rabbit	Primary	WB - 1:5000	Abcam, ab131442
phospho-eIF2α (S51)	Rabbit	Primary	WB - 1:5000	Cell Signaling Technology, 9721
phospho-gamma-H2A.X (S139), clone JBW301	Mouse	Primary	IF - 1:500	Sigma Aldrich, 05-636
phospho-gamma-H2A.X (S139)	Rabbit	Primary	IF - 1:500	Abcam, ab11174
phospho-p53 (S15)	Rabbit	Primary	WB - 1:5000	Abcam, ab1431
RIP	Rabbit	Primary	WB - 1:5000	Abcam, ab106393
RPS16	Rabbit	Primary	WB - 1:5000	Abcam, ab177951
SRSF1	Rabbit	Primary	IF - 1:500, WB - 1:5000	Abcam, ab129108
SRSF1 (103)	Mouse	Primary	IF - 1:500, WB - 1:5000	Invitrogen, 32-4600
TBP	Mouse	Primary	WB - 1:10000	Thermo Fisher Scientific, MA5-14739

Supplementary Table 5. Antibody dilutions and supplier information. WB = western blot, IHC = immunohistochemistry, IF = immunofluorescence, DB = dot blot.

Gene	Application	Forward Primer (5' > 3')	Reverse Primer (5' > 3')	Product Size
36b4	qPCR	AGATGCAGCAGATCCGCAT	GTTCTTGCCCATCAGCACC	59
Arhgef19	Splicing	CATTTCTCTGGGTCTCCCTCT	CCCACAGTCCATTCCTTCTT	148, 255
C8a	Splicing	GGTTGCTTTCTTTGGCTTGT	ACTGCAGATGGTTCCTCCAA	123, 217
Cre	qPCR	GCATTTCTGGGGATTGCTTA	ATTCTCCCACCGTCAGTACG	95
Fdxr	Splicing	CGGGTGAAGACTGTGTGGAT	GGCAGCCTCTTCCACTCC	143, 228
Gak	Splicing	TTTCCAACAGGGAAGAGCAG	CTTCCATCTCCGTCTGCAAT	124, 271
Mff	Splicing	GCAGTTGGCAGGCTAAAAAG	CTGACGTTGTCATGATGAGGAT	143, 302
Pcmtd1	Splicing	TGCCAAGGAAAAACTGGAGA	TGGACTCCAGCTCCACAGTA	150, 342
Pdk2	Splicing	TGTACTCCACAGCTCCCACA	CCTGGATGGTCTGGTAGTGG	126, 240
Pkp4	Splicing	ATAACAGGCTGCAGCACACA	ATGACTTCTGGCAGCTCAGG	121, 169
Sh3glb2	Splicing	GTGGTGGTAGGTGAGGTGCT	CACAGTGCTACCGCCACAT	118, 133
Xbp1	Splicing	ACACGCTTGGGAATGGACAC	CCATGGGAAGATGTTCTGGG	145, 171

Supplementary Table 6. Primer Sequences for qPCR and Splice Assay.

Experiment Target	Experiment Accession	Library-type	Utilized File Formats
SRSF7-human	ENCSR017PRS	paired-end	fastq, bam, bigWig
SRSF1-human	ENCSR094KBY	paired-end	fastq, bam, bigWig
SRSF9-human	ENCSR597XHH	paired-end	fastq, bam, bigWig
SRSF3-human	ENCSR376FGR	paired-end	fastq, bam, bigWig
SRSF5-human	ENCSR447UCG	paired-end	fastq, bam, bigWig
SRSF5-human	ENCSR781YNI	paired-end	fastq, bam, bigWig
Non-specific target control-human	ENCSR603TCV	paired-end	fastq, bam, bigWig
Non-specific target control-human	ENCSR264TUE	paired-end	fastq, bam, bigWig
Non-specific target control-human	ENCSR042QTH	paired-end	fastq, bam, bigWig

Supplementary Table 7. SR Protein Knockdown in HepG2 ENCODE RNA-seq details.

GEO Accession Number	Model	Genotype	Treatment	Timepoint	Replicate	Total Number of Reads
GSM4412193	SRSF1 HKO	AlbCre +/-	NA	10 days	1	101964963
GSM4412194	SRSF1 HKO	AlbCre +/-	NA	10 days	2	110546221
GSM4412195	SRSF1 HKO	SRSF1 fl/fl; AlbCre +/-	NA	10 days	1	116181095
GSM4412196	SRSF1 HKO	SRSF1 fl/fl; AlbCre +/-	NA	10 days	2	117337934
GSM4412197	SRSF1 HKO	AlbCre +/-	NA	5 weeks	1	117680056
GSM4412198	SRSF1 HKO	AlbCre +/-	NA	5 weeks	2	116619589
GSM4412199	SRSF1 HKO	SRSF1 fl/fl; AlbCre +/-	NA	5 weeks	1	115650894
GSM4412200	SRSF1 HKO	SRSF1 fl/fl; AlbCre +/-	NA	5 weeks	2	117418842
GSM4412201	acSRSF1 HKO	SRSF1 fl/fl	AAV8-TBG-GFP	2 weeks	1	127573517
GSM4412202	acSRSF1 HKO	SRSF1 fl/fl	AAV8-TBG-GFP	2 weeks	2	176265351
GSM4412203	acSRSF1 HKO	SRSF1 fl/fl	AAV8-TBG-GFP	2 weeks	3	139425979
GSM4412204	acSRSF1 HKO	SRSF1 fl/fl	AAV8-TBG-iCRE	2 weeks	1	119934148
GSM4412205	acSRSF1 HKO	SRSF1 fl/fl	AAV8-TBG-iCRE	2 weeks	2	139028073
GSM4412206	acSRSF1 HKO	SRSF1 fl/fl	AAV8-TBG-iCRE	2 weeks	3	115531464

Supplementary Table 8. SRSF1 HKO Model RNA-seq Sample Information.

Supplementary Methods

eCLIP-seq library preparation, data processing, and peak calling.

Isolated hepatocytes suspended in ice-cold 1X PBS were crosslinked with 400 mJ/cm^2 of 254 nm UV radiation to stabilize RNA binding protein (RBP)–RNA interactions. Subsequent immunoprecipitation of SRSF1-RNA complexes, RNA isolation, library preparation and sequencing were performed as described previously [1]. Briefly, crosslinked cells were lysed in buffer and sonicated, followed by treatment with RNase I (Thermo Fisher) to fragment RNA. SRSF1 antibody (A302-052A Bethyl Labs) were pre-coupled to anti-rabbit IgG Dynabeads (Thermo Fisher), added to lysate, and incubated overnight at 4 °C. Prior to IP washes, 2% of sample was removed to serve as the paired input sample. For IP samples, high- and low-salt washes were performed, after which RNA was dephosphorylated with FastAP (Thermo Fisher) and T4 PNK (NEB) at low pH, and a 3′ RNA adaptor was ligated with T4 RNA ligase (NEB). Ten per cent of IP and input samples were run on an analytical PAGE Bis-Tris protein gel, transferred to PVDF membrane, blocked in 5% dry milk in TBST, incubated with SRSF1 antibody used for IP (typically at 1:4,000 dilution), washed, incubated with HRP-conjugated anti-Rabbit secondary TrueBlot antibody (Rockland), and visualized with standard enhanced chemiluminescence imaging to validate successful IP. Ninety percent of IP and input samples were run on an analytical PAGE Bis-Tris protein gel and transferred to nitrocellulose membranes, after which the region from the protein size to 75 kDa above protein size was excised from the membrane, treated with proteinase K (NEB) to release RNA, and concentrated by column purification (Zymo). Input samples were then dephosphorylated with FastAP (Thermo Fisher) and T4 PNK (NEB) at low pH, and a 3′ RNA adaptor was ligated with T4 RNA ligase (NEB) to synchronize with IP samples. Reverse transcription was then performed with AffinityScript (Agilent), followed by ExoSAP-IT

(Affymetrix) treatment to remove the unincorporated primer. RNA was then degraded by alkaline hydrolysis, and a 3′ DNA adaptor was ligated with T4 RNA ligase (NEB). qPCR was then used to determine the required amplification, followed by PCR with Q5 (NEB) and gel electrophoresis to size-select the final library. Libraries were sequenced on the HiSeq 4000 platform (Illumina). eCLIP was performed on IP from two independent hepatocyte samples from wildtype C57BL/6j mice, along with paired size-matched input before the IP washes. eCLIP data was processed using the rigorous eCLIP processing pipeline as described previously. A detailed description of steps and scripts used is available on the ENCODE website at https://www.encodeproject.org/documents/3b1b2762-269a-4978-902e-0e1f91615782/@@download/attachment/eCLIP_analysisSOP_v2.0.pdf.

Briefly, raw eCLIP reads were demultiplexed, adapter trimmed (cutadapt v1.9.dev1), and dropped if less than 18 bp in length. Processed reads were then mapped to the mm9 mouse genome using STAR (v2.4.0i) and filtered for duplicated reads [2]. Peak calling was performed on usable reads considering only read 2 (read that is enriched for termination at crosslink site) using the publicly available tool CLIPper (available at https://github.com/YeoLab/clipper/releases/tag/1.0) with options –s mm9 –o –bonferroni –superlocal --threshold-method binomial --save-pickle [3]. Reproducible and significantly enriched peaks were identified using a modified IDR method as described previously [4]. Replicate-merged peaks with an IDR cutoff of 0.01 as well as $P \leq 0.001$ and fold enrichment ≥8 (using the geometric mean of $\log_2$(fold enrichment) between the two replicates) were considered reproducible and significant. All subsequent analysis was performed using the significant peak set.

eCLIP peak annotation and analysis.

Peak annotation was performed by overlapping peak coordinates with feature coordinates in the mouse vM19 annotation available from GENCODE. Motif enrichment analysis within SRSF1 binding peak regions was performed using the STREME function, found in The MEME Suite (v5.3.3, https://meme-suite.org/meme/index.html), using standard parameters [5]. Sequences of the binding peak regions were obtained using the getFasta function available in bedtools (2.30.0) on the peak coordinates. Gene ontology analysis was performed using Enrichr on the genes with at least one associated transcript containing at least one SRSF1 binding peak. Overlap between SRSF1 binding peaks and DEGs was defined as at least one DEG-associated transcript containing at least one SRSF1 binding peak. Overlap of SRSF1 binding peaks with differentially spliced exon was defined as the peak being within the region between the constitutive upstream and downstream exon relative to the alternative exon.

To determine if SRSF1 binding was resulting in a significant differential splicing response, the metanalysis of SRSF1 binding peak distribution was performed on differentially spliced cassette exons (SE events; n = 2,040) identified using rMATS in the acSRSF1 HKO model. The analysis approach was adapted from a previously described method [6]. Peak signals were identified for a 500 nt window flanking each exon boundary, extending a maximum of 100 nt into each exon and 400 nt into each intron. For shorter exons (<200 nt) and introns (<400 nt), the signal was counted until the boundary of the neighboring feature. This creates two windows comprising the 5' and 3' end of the cassette exon, resulting in a total vectorized region of 1000 nt. Each position within each vectorized region was marked 1 if it was within a significant peak and 0 otherwise. The values at each position were then summed and divided by the total number of events at each position to obtain the final peak density distribution. A peak distribution was also obtained for the native cassette exons (n = 2,013), which are exons found to be alternative ($0.05 < PSI < 0.95$) but do not

change significantly ($|\Delta PSI| \leq 0.05$) in acSRSF1 HKO. Finally, significance and confidence intervals were determined based on a bootstrapping approach. Peak distribution was calculated for a random sample of *n* events (without replacement) from a set of background constitutive exons (n = 23,905; PSI > 0.95 and $|\Delta PSI| \leq 0.05$), where *n* is the number of significant SE events (n = 2,040). This was repeated 1,000 times to create a distribution of peak density at each nt position. The 95% confidence interval at each position was determined to be the range bounded by the 2.5th and 97.5th percentile values. This interval was used to identify the positions where RBP-responsive event maps were significantly different from native events.

Analysis of ENCODE datasets for SR protein knockdowns.

Analyses of publicly available shRNA-seq datasets for SR proteins in HepG2 cells from the ENCODE project were performed in the same manner as described in *RNA-seq library preparation, sequencing, and analysis.* Briefly, differential gene expression analysis was performed using kallisto, tximport, and DESeq2 packages on paired-end fastq files for each sample. Differential splicing analysis was performed using rMATS on available bam alignment files. Finally, gene ontology analysis was performed using gProfiler, a web-based gene ontology analysis tool. Details regarding the datasets used for this analysis are listed in Supplementary Table 7. Briefly, ENCODE datasets of SRSF1, SRSF3, SRSF5, SRSF7, and SRSF9 protein knockdown by shRNA in HepG2 cells were used for this study [7].

Immunofluorescent staining assays in cultured cells.

For immunofluorescent staining, cells were fixed for 10 minutes using 4% PFA solution followed by a PBS wash three times and then permeabilized with 0.2% Triton X-100 plus 1% normal goat serum (NGS) in PBS/pH 7.3 for 5 minutes on ice. Cells were then washed with PBS with 1% NGS and then incubated in primary antibody dilution for 1 hour at room temperature in a humidified

chamber. This was followed by another wash and then incubation with secondary antibody dilution for 1 hour at room temperature. The cells were then washed with PBS and stained with DAPI before inverting the coverslip onto a glass slide with aqueous mounting media. All antibodies used and respective dilutions are listed in Supplementary Table 5. For the fluorescent protein synthesis assay, the Click-iT™ HPG Alexa Fluor™ 488 Protein Synthesis Assay Kit (Thermo Scientific #C10428) was used following the manufacturer's instructions. Briefly, 2 hours prior to harvesting, culture media was replaced with methionine-free DMEM media. Cells were incubated in media for 1 hour before supplementing well media with 50 μM of ClickIT-HPG reagent. Cells were incubated for an additional 1 hour before fixation. Fixed cells were prepared following standard kit protocol. For the fluorescent Annexin V cell death staining, the Dead Cell Apoptosis Kit with Annexin V FITC and PI (Thermo Scientific #V13242) was used following the manufactures instructions.

Polysome gradient fractionation.

Isolated hepatocyte samples were prepared for polysome profiling following a previously described protocol [8]. Briefly, hepatocytes were isolated as described previously; however, all buffers were supplemented with 150 μg/mL cycloheximide. The pellets were flash-frozen in liquid nitrogen and stored at -80 °C until ready for polysome fractionation. Frozen hepatocytes were thawed on ice for 15 minutes with 1 mL of polysome lysis buffer containing 10 mM Tris-HCl (pH 8.0), 150 mM NaCl, 5 mM $MgCl_2$, 1% Nonidet-P40, 40 mM dithiothreitol, 1 U/mL SUPERaseIn RNase inhibitor (Thermo Fisher) and 150 μg/mL cycloheximide. Thawed cells were pipetted gently 10 times to ensure lysis of the cytoplasm. The cell nuclei and debris were removed by centrifugation at 12,000 x g for 1 minute at 4 °C. The supernatant was transferred to a fresh tube and then centrifuged again at 16,000 x g for 7.5 minutes at 4 °C to remove remaining cell debris

and organelles. The resulting supernatant was transferred to a fresh tube, and about 400 μL supernatant was layered onto a 12 mL linear sucrose gradient (10 – 50% sucrose (w/v) made using a Biocomp Gradient Master) and centrifuged in an SW-41Ti rotor (Beckman) for 125 minutes at 38,000 r.p.m. at 4 °C. The fractionated sample was gently moved through the detector using a peristaltic pump set at 3.5 mL/min with 60% sucrose as the chase solution. Polysome profiles were measured with a UA-6 absorbance (ISCO) detector at 254 nm and recorded using the associated Peak Chart software.

Serum fractionation for lipoprotein particle analysis.

Plasma was collected from mice after a 6-hour fast starting from noon and ending at 6 p.m. Plasma (~ 200 μL) was injected into a Superose HR6 10/300 GL FPLC column (GE Healthcare). Lipoproteins were eluted with 24 mL of elution buffer containing 0.15 M NaCl, 1 mM EDTA, 0.2% w/v of sodium azide in PBS, and 0.5 mL fractions were collected at a flow rate of 0.5 mL/min. Triglyceride and cholesterol concentration was measured using Infinity kits (Thermo Scientific). In a microtiter plate, 100 μL of plasma as well as standards were incubated with 100 μL of Infinity reagent and then incubated at 37 °C for 30 minutes. The plates were measured for absorbance using a BioTek machine at 500 nm. Using absorbance measurements, the quantity of triglyceride and cholesterol was calculated.

Glucose tolerance test.

Mice were fasted overnight and injected with D-glucose at 2g/kg intraperitoneally. Blood glucose concentrations were measured at 0, 15, 30, 45, 60, and 120 min after glucose injections using the One Touch glucose meter. Blood was obtained from the tail tip after clipping.

Total RNA isolation and quantitative RT-PCR.

Total RNA was isolated from about 50 mg of snap-frozen liver tissue or snap-frozen cells from 6-well culture plates with TRIzol (Invitrogen) using the protocol described in the manual. The quality of the RNA was assessed by running about 1 µg of RNA on a bleach gel to evaluate the 28S and 18S bands [9]. Approximately 5 µg of RNA was then reverse-transcribed into cDNA using the Maxima Reverse Transcriptase (Thermo Fisher Scientific) following manufactures protocol. Relative gene expression analysis was performed with ~50 ng of cDNA per reaction using a SYBR® Green™ based assay for Real-Time Quantitative Reverse Transcription PCR (qRT-PCR) using standard cycling conditions. Using *36B4* as a loading control, relative gene expression compared to the control group was calculated by the double Ct method [10]. Primers sequences used for qPCR and splicing assays are listed in Supplementary Table 6.

Splice isoform analysis.

Total RNA and cDNA were prepared as specified as described in the section *Total RNA isolation and quantitative RT-PCR*. The cDNA was diluted to 25 ng μl^{-1} with nuclease free water from which 1 µL would be used for PCR reactions. PCR reactions were setup using Taq DNA Polymerase (NEB #M0237) along with forward and reverse primers to a final concentration of 0.2 µM. Cycling conditions used for the PCR reaction are as follows; Initial denaturation at 95 °C for 30 seconds, 30 cycles consisting of denaturation at 95 °C for 20 seconds, annealing at 60 °C for 30 seconds, and extension at 68 °C for 30 seconds, and a final extension at 68 °C for 5 minutes. For visualization, 10 µL of reaction mixture was mixed with loading dye and then resolved on a 5% PAGE gel, stained in an ethidium bromide bath, and then imaged using ChemiDoc XRS+.

TUNEL staining for apoptosis.

TUNEL staining was performed on paraffin tissue sections that were deparaffinized and rehydrated using standard procedure. Staining was performed with the *In-Situ Cell Death Detection Kit, Fluorescein* (Sigma Cat# 11684795910) using manufacturer protocol. Briefly, rehydrated tissue sections were permeabilized and then labelled using the TUNEL reaction mixture from the kit. The sections were incubated with the reaction mixture for 1 hour at 37 °C in a humidified chamber. Slides were then rinsed with PBS three times, counterstained for nucleus, and mounted with coverslip. For positive control, the section was treated with 20 U of DNAse (NEB) mix for 15 minutes. The slides were imaged using a confocal microscope.

Supplementary References

1. Van Nostrand, E. L. *et al.* Robust transcriptome-wide discovery of RNA-binding protein binding sites with enhanced CLIP (eCLIP). *Nature Methods* **13**, 508–514 (2016).
2. Dobin, A. *et al.* Sequence analysis STAR: ultrafast universal RNA-seq aligner. **29**, 15–21 (2013).
3. Lovci, M. T. *et al.* Rbfox proteins regulate alternative mRNA splicing through evolutionarily conserved RNA bridges. *Nature Structural and Molecular Biology* **20**, 1434–1442 (2013).
4. Van Nostrand, E. L. *et al.* A large-scale binding and functional map of human RNA-binding proteins. *Nature* **583**, 711–719 (2020).
5. Bailey, T. L. STREME: accurate and versatile sequence motif discovery. *Bioinformatics* (2021) doi:10.1093/bioinformatics/btab203.
6. Yee, B. A., Pratt, G. A., Graveley, B. R., van Nostrand, E. L. & Yeo, G. W. RBP-Maps enables robust generation of splicing regulatory maps. *RNA* **25**, 193–204 (2019).
7. Sundararaman, B. *et al.* Resources for the Comprehensive Discovery of Functional RNA Elements. *Molecular Cell* **61**, 903–913 (2016).
8. Seimetz, J., Arif, W., Bangru, S., Hernaez, M. & Kalsotra, A. Cell-type specific polysome profiling from mammalian tissues. *Methods* **155**, 131–139 (2019).
9. Aranda, P. S., LaJoie, D. M. & Jorcyk, C. L. Bleach gel: a simple agarose gel for analyzing RNA quality. *Electrophoresis* **33**, 366–9 (2012).
10. Livak, K. J. & Schmittgen, T. D. Analysis of relative gene expression data using real-time quantitative PCR and the 2-ΔΔCT method. *Methods* **25**, 402–408 (2001).
