# Supplementary material for: Splicing factor SRSF1 deficiency in the liver triggers NASH-like pathology and cell death
Source: Nat Commun. 2023 Feb 9;14:551. doi: 10.1038/s41467-023-35932-3 (PMC9911759; doi:10.1038/s41467-023-35932-3)
Supplement: Supplementary file 3 — Reporting Summary [file 41467_2023_35932_MOESM3_ESM.pdf]

nature portfolio

Corresponding author(s): Auinash Kalsotra

Last updated by author(s): Dec 18, 2022

Reporting Summary

Nature Portfolio wishes to improve the reproducibility of the work that we publish. This form provides structure for consistency and transparency in reporting. For further information on Nature Portfolio policies, see our Editorial Policies and the Editorial Policy Checklist.

Statistics

For all statistical analyses, confirm that the following items are present in the figure legend, table legend, main text, or Methods section.

n/a	Confirmed	
☐	☒	The exact sample size (*n*) for each experimental group/condition, given as a discrete number and unit of measurement
☐	☒	A statement on whether measurements were taken from distinct samples or whether the same sample was measured repeatedly
☐	☒	The statistical test(s) used AND whether they are one- or two-sided<br>*Only common tests should be described solely by name; describe more complex techniques in the Methods section.*
☐	☒	A description of all covariates tested
☐	☒	A description of any assumptions or corrections, such as tests of normality and adjustment for multiple comparisons
☐	☒	A full description of the statistical parameters including central tendency (e.g. means) or other basic estimates (e.g. regression coefficient) AND variation (e.g. standard deviation) or associated estimates of uncertainty (e.g. confidence intervals)
☐	☒	For null hypothesis testing, the test statistic (e.g. *F*, *t*, *r*) with confidence intervals, effect sizes, degrees of freedom and *P* value noted<br>*Give P values as exact values whenever suitable.*
☒	☐	For Bayesian analysis, information on the choice of priors and Markov chain Monte Carlo settings
☒	☐	For hierarchical and complex designs, identification of the appropriate level for tests and full reporting of outcomes
☒	☐	Estimates of effect sizes (e.g. Cohen's *d*, Pearson's *r*), indicating how they were calculated

Our web collection on statistics for biologists contains articles on many of the points above.

Software and code

Policy information about availability of computer code

Data collection	Peak chart software and FCS Express version 6 software were used to collect the polysome fractionation data. All other data collection details and are discussed in the Methods section.
Data analysis	Following softwares/scripts were used in this study: Trimmomatic (version 0.38), Kallisto (version 0.44.0), tximport (version 1.11.7), DESeq2 (version 1.23.10), STAR (version 2.4.2a), rMATS (version 3.2.5), MaxQuant (version 1.6.1.0), CLIPper (version 2.1.0), gProfiler, Enrichr, Image Lab 6.1, Cytoscape (version 3.8.0), MEME (version 5.4.1), and ggplot2 (version 3.3.3). Statistical analysis was done using Graphpad Prism 6.0h.

For manuscripts utilizing custom algorithms or software that are central to the research but not yet described in published literature, software must be made available to editors and reviewers. We strongly encourage code deposition in a community repository (e.g. GitHub). See the Nature Portfolio guidelines for submitting code & software for further information.

Data

Policy information about availability of data

All manuscripts must include a data availability statement. This statement should provide the following information, where applicable:

- Accession codes, unique identifiers, or web links for publicly available datasets
- A description of any restrictions on data availability
- For clinical datasets or third party data, please ensure that the statement adheres to our policy

The raw RNA-seq data are available for download from NCBI Gene Expression Omnibus (http://www.ncbi.nlm.nih.gov/geo/) under the accession numbers

GSE147005 (https://www.ncbi.nlm.nih.gov/geo/query/acc.cgi?acc=GSE147005) and GSE179634 (https://www.ncbi.nlm.nih.gov/geo/query/acc.cgi?acc=GSE179634).
The mass spectrometry proteomics data were deposited to the ProteomeXchange Consortium (http://proteomecentral.proteomexchange.org) via the PRIDE partner repository with the dataset identifier PXD027035 (http://proteomecentral.proteomexchange.org/cgi/GetDataset?ID=PXD027035). All source data needed to evaluate the conclusions in the paper are present in the paper and/or the Supplementary Materials.

Human research participants

Policy information about studies involving human research participants and Sex and Gender in Research.

Reporting on sex and gender	N/A
Population characteristics	N/A
Recruitment	N/A
Ethics oversight	N/A

Note that full information on the approval of the study protocol must also be provided in the manuscript.

Field-specific reporting

Please select the one below that is the best fit for your research. If you are not sure, read the appropriate sections before making your selection.

☒ Life sciences ☐ Behavioural & social sciences ☐ Ecological, evolutionary & environmental sciences

For a reference copy of the document with all sections, see nature.com/documents/nr-reporting-summary-flat.pdf

Life sciences study design

All studies must disclose on these points even when the disclosure is negative.

Sample size	Sample size of each experiment is indicated in the figure legends. For the mouse studies, 6-8 animals/genotype were used based on our previous experience to obtain statistical significance using Power analysis (e.g. G*POWER software: http://.gpower.hhu.de.en.html), which indicates >90% sensitivity for 3 biological replicates at a type 1 error of 5% when at least a 2-fold change and <20% SD of the fold change are expected. Thus, our study groups included at least twice that number of mice/time point to allow for sample size loss due to potential treatment-related mortality. For deep sequencing experiments, 2-3 biological replicates were used for each conditions.
Data exclusions	None of the data points were excluded from any of the analysis.
Replication	Cell culture experiments holding consistent results after three repetitions were considered significant. All western blots, and qPCR experiments were repeated at least three times and all attempts at replication were successful.
Randomization	Randomization was done manually. FVB/NJ and C57BL/6J strains of mice were obtained directly from the Jackson laboratories.
Blinding	Transgenic and wildtype mice were ear tagged with serial numbers, and were used to mark serum and tissue samples. Investigators were blinded to group allocation during all data collection. Samples were only identified once the final data were analyzed. NASH histopathology was scored and quantified by blinded pathologists.

Reporting for specific materials, systems and methods

We require information from authors about some types of materials, experimental systems and methods used in many studies. Here, indicate whether each material, system or method listed is relevant to your study. If you are not sure if a list item applies to your research, read the appropriate section before selecting a response.

Materials & experimental systems

n/a	Involved in the study	
☐	☒	Antibodies
☐	☒	Eukaryotic cell lines
☒	☐	Palaeontology and archaeology
☐	☒	Animals and other organisms
☒	☐	Clinical data
☒	☐	Dual use research of concern

Methods

n/a	Involved in the study	
☒	☐	ChIP-seq
☐	☒	Flow cytometry
☒	☐	MRI-based neuroimaging

Antibodies

Antibodies used	The antibody information is provided in supplementary Table 5. The Table lists the supplier and catalogue number, as well as dilutions used in all experiments. Detailed protocols are provided in the Methods section.
Validation	The validation information for all antibodies (identified by catalogue numbers in supplementary Table 5) along with relevant citations is available at the Supplier/manufacturer's website. The primary antibody species and application(s) are provided in the supplementary Table 5.

Eukaryotic cell lines

Policy information about cell lines and Sex and Gender in Research

Cell line source(s)	HepG2 cell line was directly obtained from ATCC.
Authentication	FACS analysis was performed on propidium iodide stained HepG2 cells, and it was determined that there was no contamination from a secondary cell type. Because the cells were directly authenticated by ATCC, they were not authenticated in our laboratory further.
Mycoplasma contamination	HepG2 cells were tested using staining of fixed cells with Hoechst/DAPI, and tested for presence of small mycoplasma spots around the nuclei. The cells tested negative for mycoplasma contamination.
Commonly misidentified lines (See ICLAC register)	No commonly misidentified cell lines were used in this study.

Animals and other research organisms

Policy information about studies involving animals; ARRIVE guidelines recommended for reporting animal research, and Sex and Gender in Research

Laboratory animals	Species: Mus musculus, Age: 8-10 Weeks, Sex: Male/Female.<br>Strains Used:<br>1. C57BL/6J (Jackson Laboratories, Strain #: 000664)<br>2. SRSF1 flox/flox (Jackson Laboratories, Strain #: 018020; Xu et al. (2005) Cell. 120, 59–72)<br>3. Alb-CRE (Jackson Laboratories, Strain #: 003574; Postic et al. (1999) Journal of Biological Chemistry. 274, 305–315)<br>All mice were housed in controlled temperature (18-23 degrees centigrade) and humidity (40-60%) conditions with a 12h light and dark cycle.
Wild animals	No wild animals were used in the study.
Reporting on sex	Both male and female mice were used. Sex was not considered in the study design.
Field-collected samples	No field collected samples were used in the study.
Ethics oversight	National Institutes of Health (NIH) guidelines for the use and care of laboratory animals were followed, and all experiments were approved by the Institutional Animal Care and Use Committee at the University of Illinois at Urbana-Champaign (Champaign, IL).

Note that full information on the approval of the study protocol must also be provided in the manuscript.

Flow Cytometry

Plots

Confirm that:

☒ The axis labels state the marker and fluorochrome used (e.g. CD4-FITC).

☒ The axis scales are clearly visible. Include numbers along axes only for bottom left plot of group (a 'group' is an analysis of identical markers).

☐ All plots are contour plots with outliers or pseudocolor plots.

☒ A numerical value for number of cells or percentage (with statistics) is provided.

Methodology

Sample preparation	HepG2 cells were collected by scraping and resuspended in PBS. The cells were fixed by 90% chilled ethanol overnight. Fixed cells were washed and resuspended in PBS containing 1% NGS and then incubated with 10 μg/ml of RNase A and 120 μg/ml of propidium iodine (PI) for 30 min in the dark at 37°C. The samples were passed through a cell strainer to remove clumps.
Instrument	BD LSR II analyzer
Software	FCS Express version 6
Cell population abundance	20000 events in the singlet gate were counted for each sample. The events inside the singlet gate comprised of about 66-81% of all recorded events (including debris and doublets).
Gating strategy	FSC-A vs FSC-H was used for the preliminary gating to check debris and gating for singlets were determined based on the PI-A vs PI-W plot

☒ Tick this box to confirm that a figure exemplifying the gating strategy is provided in the Supplementary Information.
